# Klinotaxis as a basic form of navigation

**DOI:** 10.3389/fnbeh.2014.00275

**Published:** 2014-08-14

**Authors:** Dominique Martinez

**Affiliations:** UMR 7503, Laboratoire Lorrain de Recherche en Informatique et ses Applications, Centre National de la Recherche ScientifiqueVandoeuvre-lès-Nancy, France

**Keywords:** klinotaxis, weathervaning, proportional navigation, *Drosophila larva*, *C. elegans*

In their article, Gomez-Marin and Louis (2014) found that runs in *Drosophila* larval chemotaxis bend toward the direction of higher concentration. This steering process called weathervaning or klinotaxis was previously discovered in the worm *C. elegans* (Ward, 1973; Iino and Yoshida, 2009) and raises the question: how is it performed? Although Gomez-Marin and Louis (2014) found that run orientation relies on the detection of the lateral gradient component, the underlying control mechanism is largely unknown. Two alternative strategies might be employed, direct error correction and proportional navigation (Figure 1A). In direct error correction, the animal would turn proportionally to the error; that is, the local bearing angle between the current heading and the desired direction to the goal (intensity peak). In proportional navigation (Murtaugh and Criel, 1966; Zarchan, 2012), the objective is to maintain a constant line of sight (LOS) angle during motion (Figure 1A). This tactic has been known by sailors for many years as a mean to detect collision courses and is currently implemented in guided missiles. Thus, in proportional navigation, the animal would turn proportionally to the derivative of the LOS angle. Here we argue that klinotaxis, as observed in Gomez-Marin and Louis (2014), does not result from direct error correction. Instead, klinotaxis is directed through proportional navigation that is a basic form of navigation (where the goal direction is estimated using gradient sensing rather than spatial memory) involving indirect error correction through an attempt to keep the LOS angle constant. Our claim is supported by theoretical analyses as well as computer simulations.

**Figure 1:**
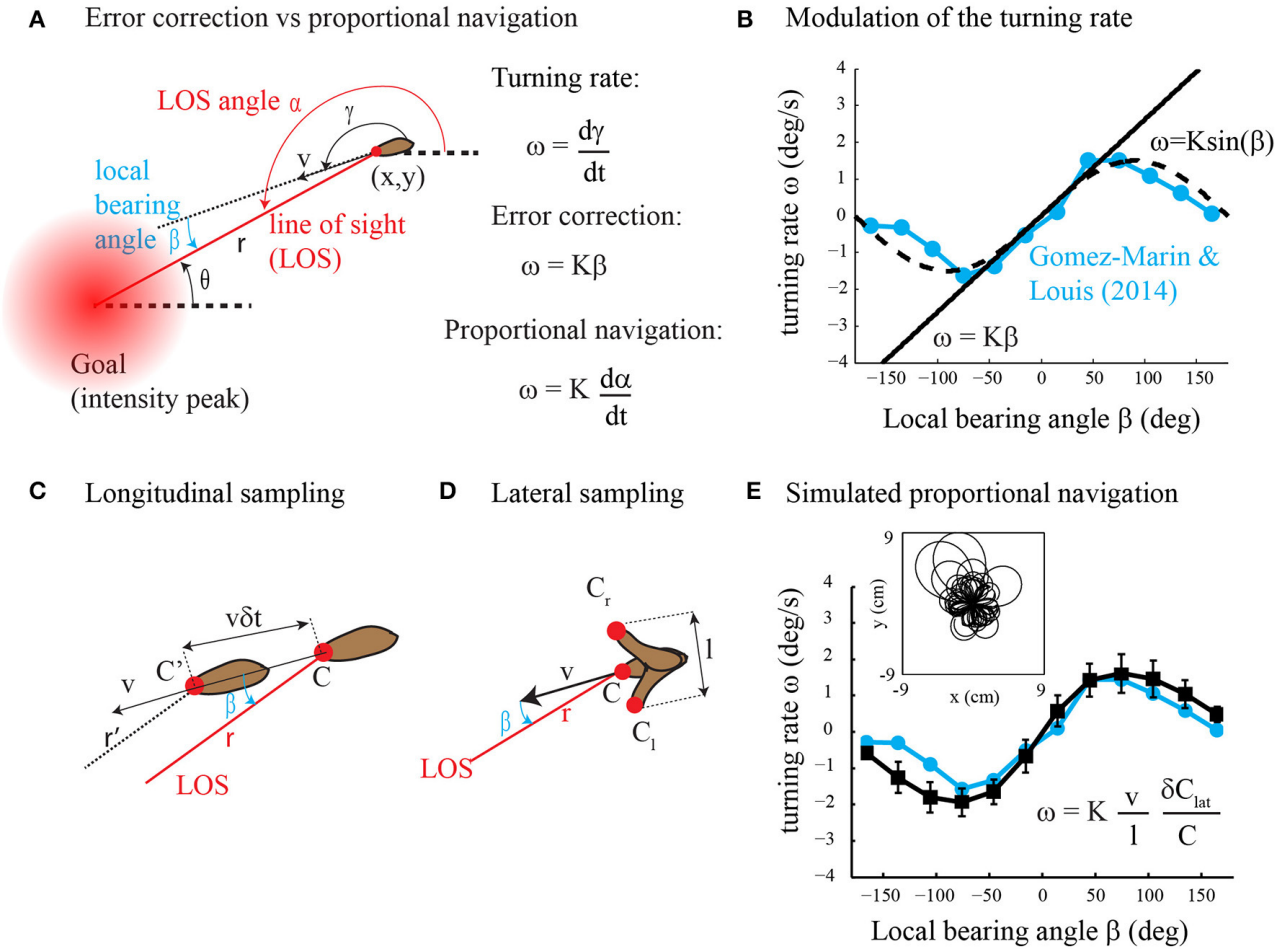
**(A)** Error correction vs. proportional navigation. The turning rate is proportional to the local bearing angle in error correction and to the rate of change of the LOS angle in proportional navigation. See text for the details. **(B)** Dependency of the turning rate on the local bearing angle. The experimental data (in blue) are reprinted with modification from Figure 1C in Gomez-Marin and Louis (2014). The straight line represents error correction (Equation 2 with *K* = 1.5). The dashed curve is for ω = *K* sin β with *K* = 1.5. **(C)** Longitudinal sampling of the gradient along the direction of motion while the animal moves forward. See text for the details. **(D)** Lateral sampling of the gradient perpendicular to the direction of motion during head sweeps. See text for the details. **(E)** Dependency of the turning rate on the local bearing angle for simulated proportional navigation (black curve, mean ± s.d., *n* = 100 trajectories) as compared to experimental data (blue curve, same as in panel **B**). The inset shows a superposition of the trajectories obtained with proportional navigation. Simulation details are as follows: the steady-state concentration field for a source that emits continuously with rate *F* = 0.1 mM/s and diffusivity *D* = 0.05 cm^2^/s is given by *C*(*r*) = *F*/(4π *Dr*). The agent starts at a random location (*x*_0_,*y*_0_) at *r*(*t* = 0) = 3 cm from the odor source. Its initial orientation is taken randomly between −π and π. The equations of motion (Equation 1 as well as ω = *d*γ/*dt*) are integrated with a time step of 0.1 s. The speed of the agent is *v* = 0.4 mm/s. The turning rate ω is calculated from Equation (7) with *K* = 3, *l* = 1 mm, δ *C*_*lat*_ = *C*_l_ −*C*_*r*_ (see panel **D**) and *C* = (*C_l_* + *C_r_*)/2. To account for intermittent and noisy observations, the relative concentration change (δ*C_lat_*/*C*) is not sampled at every time step but with 0.1 probability and multiplicative noise η(δ*C_lat_*/*C*) is added to the measurements (η = uniform random variable with mean = 0 and s.d. = 0.1).

## What kind of motor output results from direct error correction and proportional navigation?

The agent, either *Drosophila* larva or *C. elegans*, is modeled as a single point (x,y) moving at constant speed v. The equations of motion are given by

(1) $$\begin{array}{l} \begin{array}{l} \frac{dx}{dt}=v\cos\gamma \\ \frac{dy}{dt}=v\sin\gamma \end{array} \end{array}$$

where γ is the direction of movement with respect to a global reference (Figure 1A). The agent aims at orienting toward the goal by controlling its turning rate ω = *d*γ/*dt*. The orientation error is given by the local bearing angle β = α − γ where α is the line of sight (LOS) angle; that is, the absolute direction of the beeline from the agent to the goal. What kind of motor output may result from direct error correction and proportional navigation?

A direct error correction strategy would produce a motor output (turning rate ω) proportional to the error (local bearing angle β)

(2) ω=Kβ

with proportional constant K. Yet, no evidence of such a linear relationship was found in (Gomez-Marin and Louis, 2014). Instead, Figure 1B clearly shows that ω ≃ *K* sin β. A similar turning rate function is observed in *C. elegans* klinotaxis (see Figure 2A in Iino and Yoshida, 2009) and in other types of taxis, e.g., gyrotaxis (Codling et al., 2008). Can such sinusoidal reorientations be obtained with proportional navigation? Proportional navigation is a dedicated term for a basic form of navigation that aims at maintaining the LOS angle α constant during motion (Murtaugh and Criel, 1966; Zarchan, 2012). It was originally developed for missile guidance toward moving targets. Recently, it has been employed for navigating a robot toward a fixed goal with the kinematics given by Equation (1) and was defined as follows (Belkhouche and Belkhouche, 2007).

(3) $$\omega =K\frac{d\alpha }{dt}$$

so that the agent moves in a straight line when α remains constant. In the Supplementary Materials, we show that the rate of change of α is given by *v* sin β/*r* so that

(4) $$\omega =K\frac{v}{r}\sin\beta$$

Thus, proportional navigation is expected to produce a motor output ω proportional to the sine of β, similar to that observed experimentally (Figure 1B). Note however that Equation (4) is useless in practice as both the distance *r* to the goal and local bearing angle β are unknown to the agent. The sensory input required for proportional navigation is examined in the next section.

## What kind of sensory information is needed for direct error correction and proportional navigation?

*Drosophila* larva and *C. elegans* assess the chemical gradient by comparing the stimulus intensity over time. Yet, it is not known exactly how the gradient is sampled. It could be sampled along the direction of motion while the animal moves forward (Ferrée and Lockery, 1999) or perpendicular to the direction of motion during head sweeps (Ward, 1973; Iino and Yoshida, 2009; Gomez-Marin and Louis, 2014). The *Drosophila* larva moves at *v* ≃ 0.5–1 mm/s and may integrate information on time scales of a few seconds, and low-amplitude head sweeps lead to side-by-side deflections of the head of *l* ≃ 0.7 mm (Gomez-Marin and Louis, 2014). Thus, measurements along or perpendicular to the direction of motion would be performed over distances of about 1 mm. Applying the same logic as in Gomez-Marin et al. (2010), we estimate that concentration differences are 10-fold larger than the noise level so that both lateral and longitudinal samplings are viable options. In what follows, we therefore investigate the two possibilities.

In Figure 1C, we consider that the agent takes measurements every δ*t* sec during forward locomotion. The longitudinal gradient component is estimated along the direction of motion as δ*C_lon_*/(*v*δ*t*) where δ*C_lon_* is the measured difference in concentration and *v*δ*t* is the distance traveled between two consecutive measurements. In the Supplementary Materials, we show that the longitudinal gradient component is related to the local bearing angle as follows.

(5) $$\cos\beta ≃\frac{r}{C}\left(\frac{\delta C_{lon}}{v\delta t}\right)$$

In Figure 1D, we consider side-to-side movements of the head sensor of amplitude *l* perpendicular to the direction of motion so that the lateral gradient component is estimated as δ*C_lat_*/*l* where δ*C_lat_* is the difference in concentration from one side to the other. In the Supplementary Materials, we show that the lateral gradient component is related to the local bearing angle as follows.

(6) $$\sin\beta ≃\frac{r}{C}\left(\frac{\delta C_{lat}}{l}\right)$$

How to make use of this sensory information? From Equation (2), error correction requires the value of β. Taking the inverse cosine of Equation (5) leads to ±β whereas taking the inverse sine of Equation (6) leads to β or π −β. These ambiguities prevent the use of either the lateral or the longitudinal gradient component alone. In contrast, an efficient implementation of Equation (2) using both longitudinal and lateral gradient components is provided by ω = *K* atan2(sinβ, cosβ) where atan2 is the four-quadrant inverse tangent.

From Equation (4), proportional navigation requires the value of sinβ. On the one hand, the use of Equation (5) leads to $\beta =\pm \sqrt{1-\cos^{2}\beta }$ which makes the turning direction unknown. On the other hand, the lateral gradient component alone is sufficient for proportional navigation. From Equations (4) and (6), proportional navigation writes as follows.

(7) $$\omega =K\frac{v}{l}\left(\frac{\delta C_{lat}}{C}\right)$$

We note that the turning rate ω =*d*γ/*dt* in Equation (7) is proportional to the relative change in stimulus intensity δ*C_lat_*/*C* so that proportional navigation obeys the Weber-Fechner law (Kandel et al., 2000). Figure 1E shows numerical results of Equation (7) (simulation details in Figure caption) that are in good agreement with experimental data. We also note that the robotic implementation of Equation (7) appeared to be very robust in real conditions (see illustrative video at http://youtu.be/XvVERq4h_uc).

From experimental studies (Iino and Yoshida, 2009; Gomez-Marin and Louis, 2014), we know that the relationship between turning rate and bearing error in *Drosophila* larva and *C. elegans* is approximately sinusoidal. Also, in both animals, the turning rate is clearly correlated with the lateral gradient component and not with the longitudinal gradient component. Here, we show that proportional navigation produces a sinusoidal relationship between turning rate and bearing error and merely employs the lateral gradient component. In contrast, direct error correction is linear *per se* and requires both lateral and longitudinal gradient components. Together, these data suggest that klinotaxis reflects proportional navigation rather than direct error correction. Nevertheless, because it is fairly impossible to rule out that another model may explain the data equally well, the conclusion that klinotaxis is directed through proportional navigation remains a viable hypothesis in the absence of compelling experimental evidence.

Proportional navigation is a strategy employed by certain predators to track unpredictably moving targets, e.g., bats chasing insects (Ghose et al., 2006). In a prey pursuit, proportional navigation results in an advantage for the pursuer that is known as motion camouflage (Mizutani et al., 2003). What could be the interest of proportional navigation for klinotaxis? At first sight, the sine relationship between turning rate and bearing error does not seem optimal because small turning rates are produced for large bearing errors. However, the continuity of the sinus with zero crossing at ±180° may prevent chaotic reorientations in the presence of noise. In addition, proportional navigation (unlike direct error correction) requires minimal sensory information; i.e., the lateral gradient component. Thus, *Drosophila* larvae and *C. elegans* may prefer proportional navigation over direct error correction because of its simplicity and robustness, just like proportional navigation is the preferred method of missile guidance because it provides the best performance with minimal sensory information (Murtaugh and Criel, 1966; Zarchan, 2012).

### Conflict of interest statement

The author declares that the research was conducted in the absence of any commercial or financial relationships that could be construed as a potential conflict of interest.
