# Supplementary material for: Klinotaxis as a basic form of navigation
Source: Front Behav Neurosci. 2014 Aug 14;8:275. doi: 10.3389/fnbeh.2014.00275 (PMC4132367; doi:10.3389/fnbeh.2014.00275)
Supplement: Supplementary file 1 [file DataSheet1.PDF]

## APPENDIX

### Rate of change of the LOS angle as a function of β

To derive the rate of change of the LOS angle α as a function of the local bearing angle β (Figure 1A), it is convenient to rewrite the equations of motion $dx/dt = v\cos\gamma$ and $dy/dt = v\sin\gamma$ in polar coordinates using the change of variables:

$$x = r\cos\theta$$

$$y = r\sin\theta \tag{S1}$$

The angular speed writes:

$$\frac{d\theta}{dt} = \frac{x\,dy/dt - y\,dx/dt}{r^2} \tag{S2}$$

Using Eq. S1 and the equations of motion, Eq. S2 becomes:

$$r\frac{d\theta}{dt} = v(\cos\theta\sin\gamma - \sin\theta\cos\gamma)$$

$$= v\sin(\gamma - \theta)$$

As $\alpha = \theta + \pi$ (Figure 1A), the rate of change of the LOS angle is $\frac{d\alpha}{dt} = \frac{d\theta}{dt}$ and is given by:

$$r\frac{d\alpha}{dt} = -v\sin(\gamma - \alpha)$$

Using the relation $\beta = \alpha - \gamma$, one finally obtains:

$$r\frac{d\alpha}{dt} = v\sin\beta$$

### Longitudinal gradient component as a function of β

We consider that the agent moves at constant speed $v$ and takes two concentration measurements $C$ and $C'$ at time $t$ and $t + \delta t$, respectively (Figure 1C). During the sampling time, the traveled distance is $v\delta t$ and the LOS distance changes from $r$ to $r'$. The LOS distance at time $t + \delta t$ can be written as:

$$r' = \sqrt{r^2 + v^2\delta t^2 + 2rv\delta t\cos(\theta - \gamma)}$$

From Figure 1A, we see that $\theta + \pi = \gamma + \beta$. Thus, $\cos(\theta - \gamma) = \cos(\beta - \pi) = -\cos\beta$ leading to:

$$r' = r\sqrt{1 + \frac{v^2\delta t^2}{r^2} - \frac{2v\delta t}{r}\cos\beta} \tag{S3}$$

Sufficiently far from the source ($r \gg v\delta t$), the second order term in Eq. S3 can be neglected. Thus, Eq. S3 simplifies as follows.

$$r' \simeq r\sqrt{1 - \frac{2v\delta t}{r}\cos\beta} \tag{S4}$$

For a source that emits continuously with rate F and diffusivity D, the concentration at time $t$ and distance $r$ is (Berg, 1993):

$$C(r,t) = \int_0^t \frac{F}{(4\pi D t')^{3/2}} e^{-r^2/4Dt'} dt'$$

If diffusion lasts long enough, the concentration approaches the steady-state value $C = a/r$ with $a = F/(4\pi D)$ (Berg, 1993). Such concentration fields, inversely proportional to the distance from the source, have been confirmed experimentally in behavioral assays (Ohashi et al. 2014). Thus, at distance $r'$, one has $C' = a/r'$ with $r'$given by Eq. S4. For $r \gg v\delta t$, we may simplify the expression by writing $1/\sqrt{1-\varepsilon} \simeq 1 + \varepsilon/2$ so that:

$$C' \simeq C\left(1 + \frac{v\delta t}{r}\cos\beta\right)$$

The concentration difference $\delta C_{lon} = C' - C$ is finally given by:

$$\delta C_{lon} \simeq \frac{C}{r} v\delta t \cos\beta$$

**Lateral gradient component as a function of β**

We consider that the animal moves at constant speed $v$ and takes two concentration measurements $C_r$ and $C_l$ perpendicular to the direction of motion (Figure 1D). Using a derivation similar to the one in section 2, we find:

$$C_r \simeq C\left(1 - \frac{l}{2r}\sin\beta\right)$$
$$C_l \simeq C\left(1 + \frac{l}{2r}\sin\beta\right)$$

with the approximation valid sufficiently far from the source ($r \gg l$). The concentration difference $\delta C_{lat} = C_l - C_r$ is finally given by:

$$\delta C_{lat} \simeq C\frac{l}{r}\sin\beta$$

## References


Berg, H.C. (1993) Random Walks in Biology, Princeton University Press, Princeton, New Jersey.

Ohashi, S., Morimoto, T., Suzuki, Y., Miyakawa, H., Aonishi, T. (2014). A novel behavioral strategy, continuous biased running, during chemotaxis in Drosophila larvae. Neurosci Lett. 570:10-5. doi: 10.1016/j.neulet.2014.04.011.
